# Un cas d’angiosarcome de l’oreille externe

**DOI:** 10.11604/pamj.2016.24.177.9834

**Published:** 2016-06-30

**Authors:** Karima Issara, Zouhour Boughaleb, Nezha Tawfiq, Zineb Bouchbika, Nadia Benchakroun, Hassan Jouhadi, Souha Sahraoui, Abdellatif Benider

**Affiliations:** 1 Centre Mohammed VI de Traitement des Cancers, CHU Ibn Rochd, Faculté de Médecine et Pharmacie, Université Hassan II, Casablanca, Maroc

**Keywords:** Angiosarcome, oreille, peau, radiothérapie, chimiothérapie, Angiosarcoma, ear, skin, radiotherapy, chemotherapy

## Abstract

L’angiosarcome de l’oreille est une tumeur maligne très rare, et localement très agressive. Elle représente 4-5% des sarcomes cutanés et moins de 1% de tous les sarcomes. Notre objectif est de décrire et de discuter les modalités diagnostiques et thérapeutiques de cette tumeur maligne. A travers le cas clinique d’une patiente âgée de 31 ans reçue pour une masse bourgeonnante du pavillon de l’oreille gauche. Le scanner était en faveur d’une tumeur du pavillon d’oreille avec envahissement de la parotide homolatérale. Le diagnostic histologique était en faveur d’un angiosarcome. Le traitement a consisté en une chirurgie d’exérèse complète suivie d’une radiothérapie adjuvante. Elle est en rémission compléte avec un recul d’une année.

## Abstract (en)

Angiosarcoma of the ear is a very rare and locally aggressive malignant tumor. It accounts for 4-5% of cutaneous sarcomas and less than 1% of all sarcomas. Our study aims to describe and discuss diagnostic and therapeutic modalities of this malignancy. We report the clinical case of a 31 year old patient with bulging mass of the pavilion of the left ear. CT scan was in favor of tumor of the pavilion of the ear invading ipsilateral parotid. Histological diagnosis was in favor of angiosarcoma. The treatment consisted of complete surgical resection followed by adjuvant radiotherapy. She is in complete remission with a follow-up period of 1 year.

## Introduction

Les tumeurs malignes de l’oreille sont des tumeurs rares, avec une incidence d’un cas pour six millions d’habitants. Les angiosarcomes cutanés sont rares, et représentent 4-5% des sarcomes cutanés et moins de 1% de tous les sarcomes. La localisation de ces tumeurs à l’oreille externe est exceptionnelle [1]. Elle est surtout observée chez les personnes âgées, les hommes étant deux fois plus touchés que les femmes [2]. Le pronostic est péjoratif avec une médiane de survie passant de 7 mois sans traitement à 20 mois après chirurgie et radiothérapie [3, 4]. A travers notre observation, nous allons décrire et discuter les aspects diagnostiques et thérapeutiques de l’angiosarcome du pavillon de l’oreille.

## Patient et observation

Une patiente âgée de 31 ans, sans aucun antécédent pathologique particulier, a été reçue en consultation pour une masse du pavillon de l’oreille externe gauche évoluant progressivement depuis un an auparavant.

Sur le plan clinique, la patiente présentait une masse bourgeonnante au dépend de la partie inférieure du pavillon de l’oreille gauche mesurant 6 cm de grand diamètre, ferme, indolore, sans signes inflammatoires et sans paralysie faciale périphérique associée. La tomodensitométrie avait objectivé une tumeur du pavillon de l’oreille gauche, bien limitée, mesurant 48 mm de grand axe et étendue sur 27 mm, envahissant la parotide homolatérale et arrivant au contact de la mastoïde homolatérale sans perte du liseré graisseux de séparation.

**Figure 1:**
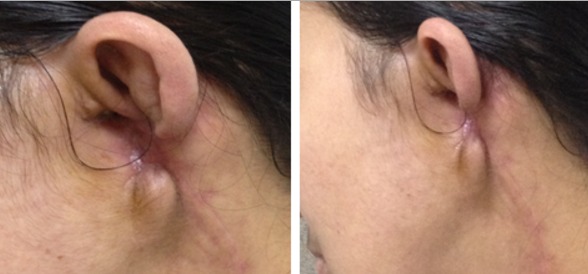
Aspect clinique post chirurgical

Un scanner thoracique, réalisé dans le cadre du bilan d’extension, était normal. Le traitement a consisté en une chirurgie large incluant l’exérèse du lobule de l’oreille gauche avec une recoupe profonde interne du planché du conduit auditif externe, associée à une parotidectomie superficielle gauche. L’examen histopathologique de la pièce opératoire a objectivé une tumeur exophytique, hémorragique, friable mesurant 6/7 cm. A l’étude histologique il s’agissait d’une prolifération sarcomateuse faite de cellules fusiformes, des cavités vasculaires et des atypies cytonucléaires en faveur d’angiosarcome moyennement différencié de haut grade du pavillon de l’oreille infiltrant le muscle et le cartilage sans emboles néoplasiques. Les marges de résection étaient saines; la plus proche étant à 5 mm. La recoupe profonde était saine. La pièce de parotidectomie a montré un parenchyme glandulaire lobulé et dissocié par une fibrose collagène, contenant des amas de cellules inflammatoires avec la présence de ganglions lymphoïdes d’aspect réactionnel. Les suites post opératoires ont étaient simples (Figure 1).

**Figure 2:**
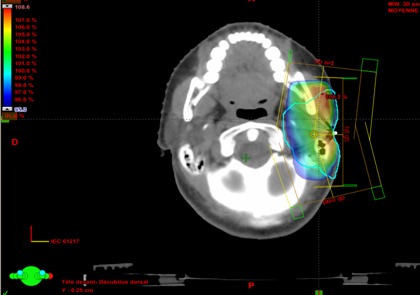
Distribution de la couverture du volume cible à irradier par la 95% de la dose prescrite

Une radiothérapie conformation elle tridimensionnelle locale adjuvante a été prescrite pour assurer le contrôle local. Le lit tumoral a reçu une dose de 60 Gy en 30 fractions de 2 Gy, 5 séances par semaine par des faisceaux obliques antérieurs, oblique postérieur et latéral gauche. La Figure 2 comporte une image de la dosimétrie.

La patiente est en rémission complète clinique et radiologique, avec un recul actuel d’une année.

## Discussion

Les tumeurs malignes de l’oreille sont peu fréquentes avec une incidence d’un cas pour six millions d’habitants, quelque soit le type histopathologique. Les angiosarcomes cutanés sont rares, et représentent 4-5% des sarcomes cutanés et moins de 1% de tous les sarcomes. La localisation de ces tumeurs à l’oreille externe est exceptionnelle [1]. Elle est surtout observée chez les personnes âgées, les hommes étant deux fois plus touchés que les femmes [2].

Certains facteurs sont connus: un lymphoedème chronique, congénital ou traumatique, est impliqué dans plus de 10% des angiosarcomes des membres; les radiations ionisantes; ils se produisent le plus souvent dans les zones de la peau exposée à long terme au soleil chez les patients âgés; exceptionnellement, les angiosarcomes peuvent se développer sur un angiome pré existant [3].

La présentation clinique de cette tumeur rare est variable, avec des ecchymoses mal définies ou des zones d’érythème, puis apparition de plaques indurées avec des éléments nodulaires ou ulcérés, cette variabilité donne souvent confusion avec des lésions bénignes et retarde le diagnostic. Plusieurs études rétrospectives confirment la difficulté du diagnostic clinique de l’angiosarcome cutané [1, 3].

Sur le plan histologique, les angiosarcomes vont d’une tumeur hautement différenciée ressemblant à un hémangiome, à une tumeur anaplasique difficile à distinguer d’un carcinome [1]. Les formes moyennement et bien différenciées sont caractérisées par la présence de cavités vasculaires irrégulières, qui dissèquent le collagène et réalisent un réseau anastomotique, bordé de cellules à noyaux augmentés de volume et souvent hyper chromatiques [3].

Le bilan d’extension fait recours à une IRM cervico-faciale à la recherche d’extension locorégionale, et une TDM thoraco-abdominale à la recherche d’extension à distance [2].

Le traitement de l’angiosarcome repose sur la chirurgie radicale suivie d’une radiothérapie adjuvante. L’exérèse chirurgicale doit être la plus large possible avec des marges de 2 à 5 cm en restant compatible avec une qualité de vie acceptable [2]. Cependant, de larges marges chirurgicales sont souvent difficiles à obtenir vu la localisation de ces tumeurs; dans la plupart des séries, seules les petites tumeurs (< 5cm), de bas grade et avec une résection complète microscopique peuvent bénéficier d’une chirurgie seule [5]. L’association de la radiothérapie à la chirurgie offre de meilleures chances de contrôle local par rapport à une chirurgie seule, même après une résection complète [4]. Dans une série de 28 patients traités pour un angiosarcome de la tête et du cou, la survie médiane à 32 mois était meilleure pour la combinaison chirurgie et radiothérapie par rapport à une chirurgie seule [6, 7]. Une association de radio chimiothérapie concomitante est une alternative à la chirurgie, si une résection complète s’avère impossible. Fujisawa et al (2014 la date doit être mise dans la discussion) rapporte une prolongation de la survie chez un groupe de patients traités par une radio chimiothérapie concomitante suivie de chimiothérapie adjuvante à base de taxanes par rapport au groupe de patients ayant été traités par une chirurgie suivie de radiothérapie [8]. Des études plus larges sont nécessaires.

L’angiosarcome est responsable d’une évolution métastatique dans 50% des cas, considéré ainsi comme une maladie systémique. Il n’y a pas de données cohérentes à l’appui de l’administration de la chimiothérapie adjuvante pour ces tumeurs [9]. Au stade métastatique, par analogie avec d’autres sous types histologiques de sarcomes de tissus mous, le traitement classique est basé sur un protocole de chimiothérapie à base de doxorubicine [9, 10]. En outre, certaines études rétrospectives [9, 11] et un essai de phase II [12] suggèrent le paclitaxel hebdomadaire comme une option alternative intéressante pour l’angiosarcome métastatique.

Le pronostic de l’angiosarcome est péjoratif; chez les patients non traités la survie médiane est de 7 mois, passant à 20 mois après un traitement par chirurgie et radiothérapie; avec une survie globale à 5 ans de 12% [3, 4].

## Conclusion

A travers ce travail, nous rapportons un cas rare d’un angiosarcome cutané localisé au niveau du pavillon de l’oreille. Un retard de diagnostic pourrait aboutir à des défis de traitement importants. Dans notre cas, une chirurgie complète a pu être réalisée complétée par une radiothérapie adjuvante, avec une bonne réponse clinique.
